# Diagnostic Accuracy of Cystic Lesions Using a Pre-Programmed Low-Dose and Standard-Dose Dental Cone-Beam Computed Tomography Protocol: An Ex Vivo Comparison Study

**DOI:** 10.3390/s21217402

**Published:** 2021-11-07

**Authors:** Adib Al-Haj Husain, Quirin Döbelin, Barbara Giacomelli-Hiestand, Daniel B. Wiedemeier, Bernd Stadlinger, Silvio Valdec

**Affiliations:** 1 Clinic of Cranio-Maxillofacial and Oral Surgery, Center of Dental Medicine, University of Zurich, 8032 Zurich, Switzerland; adib.al-hajhusain@uzh.ch (A.A.-H.H.); quirin.doebelin@hispeed.ch (Q.D.); info@praxis-giacomelli.ch (B.G.-H.); bernd.stadlinger@zzm.uzh.ch (B.S.); 2 Statistical Services, Center of Dental Medicine, University of Zurich, 8032 Zurich, Switzerland; daniel.wiedemeier@zzm.uzh.ch; 3 Department of Stomatology, Division of Periodontology, Dental School, University of São Paulo, Butantã, São Paulo 2227, Brazil

**Keywords:** cone-beam computed tomography, low-dose cone-beam computed tomography, low dose protocols, cystic lesion, oral surgery, oral anatomy

## Abstract

Background: This study aimed to analyze the diagnostic reliability of radiographic assessment of cystic lesions using a pre-set, manufacturer-specific, low-dose mode compared to a standard-dose dental cone-beam computed tomography (CBCT) imaging protocol. Methods: Forty pig mandible models were prepared with cystic lesions and underwent both CBCT protocols on an Orthophos SL Unit (Dentsply-Sirona, Bensheim, Germany). Qualitative and quantitative analysis of CBCT data was performed by twelve investigators independently in SIDEXIS 4 (Dentsply-Sirona) using a trial-specific digital examination software tool. Thereby, the effect of the two dose types on overall detectability rate, the visibility on a scale of 1 (very low) to 10 (very high) and the difference between measured radiographic and actual lesion size was assessed. Results: Low-dose CBCT imaging showed no significant differences considering detectability (78.8% vs. 81.6%) and visibility (9.16 vs. 9.19) of cystic lesions compared to the standard protocol. Both imaging protocols performed very similarly in lesion size assessment, with an apparent underestimation of the actual size. Conclusion: Low-dose protocols providing confidential diagnostic evaluation with an improved benefit–risk ratio according to the ALADA principle could become a promising alternative as a primary diagnostic tool as well as for radiological follow-up in the treatment of cystic lesions.

## 1. Introduction

Clinical and radiological diagnosis of a cystic mass in the jaw is a daily challenge in dental surgery, especially when various differential diagnoses are suspected. Cystic and cystic-appearing lesions may be of odontogenic or nonodontogenic origin, mineralized or nonmineralized, and can range from benign indolent to invasive malignant tumors [1]. Since many lesions have similar radiologic features, differentiation based on radiological appearance alone is not possible; therefore, biopsy is required in addition to clinical examination for final diagnosis [2]. The type of cyst significantly influences therapy, but both complete enucleation of the cyst (cystectomy) and marsupialization to the oral cavity (cystostomy) require radiological follow-up in some cases over many years to assess the extent of the bony lesion or neo-ossification [3,4].

In recent years, biomedical imaging has made tremendous progress and opened many possibilities for preoperative diagnostics, particularly in dentoalveolar surgery, leading to improved personalized therapy options and thus, better clinical outcomes [5,6]. Taking into account the increased controversy over the need to optimize the radiation dose in accordance with the “as low as reasonably achievable” (ALARA) principle and the recently proposed paradigm shift to the “as low as diagnostically acceptable” (ALADA) principle [7], some promising modifications have recently been tested, such as the use of low-dose cone-beam computed tomography (CBCT) imaging protocols or magnetic resonance imaging (MRI) in the dentomaxillofacial field [8,9,10,11,12]. Although recently, published reports highlight the enormous potential for development, there are still some significant limitations, so MRI is not yet the most commonly used and particularly indicated imaging modality in clinical practice [13,14]. Another interesting non-invasive imaging modality for detecting bony lesions in the jaws is ultrasound, as it offers better detectability of lesions than conventional radiation-based imaging. Nevertheless, there are still concerns about its reliability and practicability in everyday clinical practice [15].

In clinical practice, conventional radiation-based panoramic radiography (PAN) is often routinely used as a primary diagnostic tool for the detection of cystic lesions, particularly in follow-up to assess neo-ossification. In more complex maxillofacial surgical cases requiring three-dimensional information of the region of interest, CBCT offers advantages over conventional 2D imaging modalities, such as superior detectability of osseous lesions in the bucco-oral dimension and the morphology of osseous defects such as dehiscence or fenestration [16,17]. However, the assessment of the precise bone density of the jaw using CBCT, an essential parameter in various oral and maxillofacial procedures, e.g., implantation, should be considered with caution as reports indicate that the assessment of bone density is not useful if the values are taken as absolute values [18].

Compared to conventional computed tomography (CT), CBCT generally has markedly reduced radiation exposure, which can be further decreased by the use of low-dose protocols [19]. Despite insufficient soft tissue contrast and standardized grayscale values, CBCT is considered the gold standard in computer-assisted oral and maxillofacial surgery due to higher accessibility and lower costs [20,21]. Despite the advantage of providing added surgically relevant information from CBCT use [22], the main disadvantage is the radiation exposure of approximately 18–200 µS per examination, which is particularly relevant for repeated thyroid radiation exposure in the highly radiosensitive group of younger patients, resulting in increased lifetime risk of radiation-induced cancer [23,24,25]. Therefore, it is elementary to understand the indications and limitations of radiographic CBCT examinations, which should be performed only when they provide additional diagnostic value that cannot be obtained with two-dimensional imaging modalities with lower radiation doses [26]. Hence, the diagnostic reliability of low-dose CBCT imaging protocols should be evaluated, determining the unique indications and limitations for case-specific clinical questions considering the ALARA and ALADA principles [27,28].

The effective radiation dose depends on many factors, including the field of view (FOV), image resolution, size of the patients, region of interest (ROI), and manufacturer-specific scan parameters. Significant radiation exposure optimization can be achieved by reducing the FOV, ROI, by adjustment of tube voltage (kV) and determining the minimally acceptable tube current (mA) [8]. Considering the large number of CBCT scanner manufacturers and the associated heterogeneity of scanner-specific parameters, even standardized low-dose protocols should always be modified and optimized individually [26,29]. Despite the altered image resolution and image noise due to tube voltage and tube current adjustments [26], previous reports have shown that low-dose CBCT imaging protocols present a promising diagnostic tool in many clinical settings [7,9,29,30].

As pre-set, manufacturer-specific low-dose imaging protocols provide a user-friendly option for predictable image quality, this study aimed to investigate the diagnostic accuracy in the radiographic assessment of mandibular cystic lesions compared to a standard-dose dental CBCT imaging protocol and to identify potential discrepancies in detection rates between low-dose and standard-dose protocols. However, for ethical and safety reasons, these studies cannot be conducted in vivo [27]. Therefore, pig mandibles, a suitable and common animal model in dental research [31], were used for this ex vivo comparative study.

## 2. Materials and Methods

### 2.1. Preparation of Cystic Lesions

**Figure 1:**
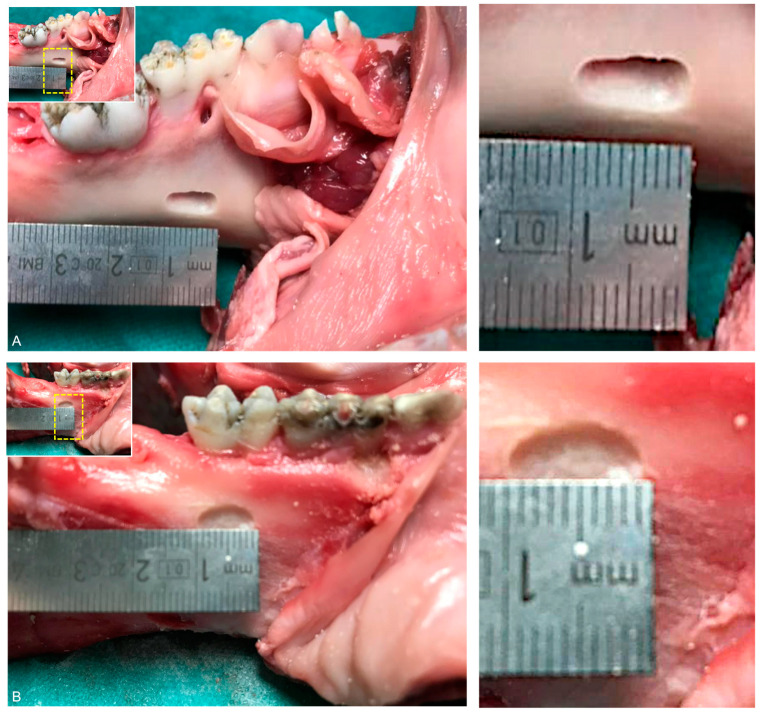
Special drilling instruments (rose head bur H141, Komet Dental, DENTAL Brasseler GmbH, Lemgo, Germany; handpiece Kavo Expertmatic E10C, KaVo Dental AG, Kloten, Switzerland) were used to prepare (**A**) lingual and (**B**) buccal mandibular cystic lesions of the same size after partial removal of soft tissue and epithelium and underwent standard and low-dose CBCT imaging protocols.

In this study setting, 40 mandibles of pig cadavers were obtained from the local slaughterhouse in Zurich, Switzerland. Special drilling instruments (rose head bur H141, Komet Dental, DENTAL Brasseler GmbH, Lemgo, Germany; handpiece Kavo Expertmatic E10C, KaVo Dental AG, Kloten, Switzerland) were used to simulate mandibular cystic lesions of the same size after partial removal of soft tissue and epithelium (Figure 1). This procedure was repeated in all mandibles, in randomized order. A declaration of non-responsibility from the cantonal veterinary services was obtained from the Office of Animal Welfare and 3R of the University of Zurich. Hence, all experiments comply with the policy of the University of Zurich on animal experimental Research.

### 2.2. CBCT Data Acquisition

All mandibles underwent low-dose and standard-dose CBCT imaging protocol at a FOV of 11 × 10 cm. The applied low-dose CBCT imaging protocol with an effective radiation dose of 20 µSv had the following sequence specifications: 85 kV; 13 mA; exposure time 2.2 s and pixel size 0.160 mm, whereby the standard imaging protocol with an effective dose of 145 µSv contained the following parameters: 85 kV; 13 mA; radiation time 4.4 s and voxel size 160 μm [32] (Table 1). To imitate the in vivo conditions as closely as possible, the soft tissue was simulated with a cold pack (12 × 29 cm, GELLO Geltechnik GmbH, Ahaus, Germany) in the center of the mandible. A total of 80 CBCT scans (40, low-dose protocol; 40, standard-dose protocol) were performed.

### 2.3. Image Evaluation

Storage and analysis of the CBCT DICOM data were performed in a modified version of the Sidexis 4 Software (Dentsply Sirona, York, PA, USA) using the same workstation (Supermicro, Windows 10 Professional Edition 64 Bit; Intel^®^ Core™ i7-6700 CPU © 3.40 GHz Intel64 Family 6 Model 94 Stepping 3, 3400 MHz/x64) and display (HP Z23n 58, 4 cm, 23 Inch, IPS LED Backlight). Eighty CBCT scans were randomly assigned into 16 groups of five CBCT scans each and evaluated independently by 12 investigators (eight oral surgeons, four maxillofacial surgeons) with a minimum of two years of training in oral radiology and the diagnosis of CBCT images. Prior to evaluation, a calibration session was conducted in which each examiner received instructions from one of the principal investigators (Q.D.), and five randomly selected cases were evaluated to eliminate ambiguities. All readers were blinded to each other’s results. The examination included the following steps: first, the cystic lesion was marked with a cursor, second, the qualitative analysis of the visibility of the lesion was assessed using a scale from 1 (very low) to 10 (very high), and third, the difference between the measured and actual size of the lesion at its greatest extent was examined, considering only those measurements that were detected and measured by the evaluator in both protocols. A digital coordinate system on the Sidexis 4 software was used to perform the measurements, and all data were collected in an Excel spreadsheet (Microsoft Excel 2020, Microsoft Corporation, Redmond, WA, USA).

### 2.4. Statistical Analysis

A mixed-effects logistic regression was fitted to the data to assess the differences in the detection of cystic lesion using low-dose and standard-dose protocols. The target variable (correct detection) was modelled by the fixed variable (radiation dose) using random intercepts to account for the repeated measures design (examiner). The model fit was inspected using residual analysis and did not show relevant violations of model assumptions. The detectability of cystic lesions for each radiation dose was thus estimated from the model using marginal means and tested using log odds.

To investigate whether the differences between the measured and actual lesion size depended on the imaging protocol, these differences were compared using a Wilcoxon signed-rank test (because parametric assumptions were violated).

All statistical analyses were performed using the statistical software R 4.0.5 [33], including the packages ImerTest [34], emmeans [35], and DHARMa [36] and a significance level α = 0.05 was used for the hypotheses tests.

## 3. Results

**Figure 2:**
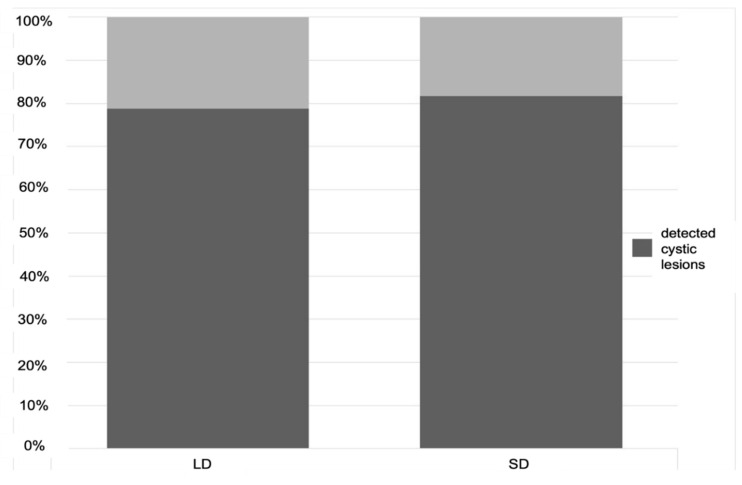
Detectability rates (percentage) of the cystic lesions in the low dose (LD) and standard-dose (SD) imaging protocol. The detection of cystic lesions was successful in 78.8% of cases with the low-dose protocol and in 81.6% with the standard protocol.

**Figure 3:**
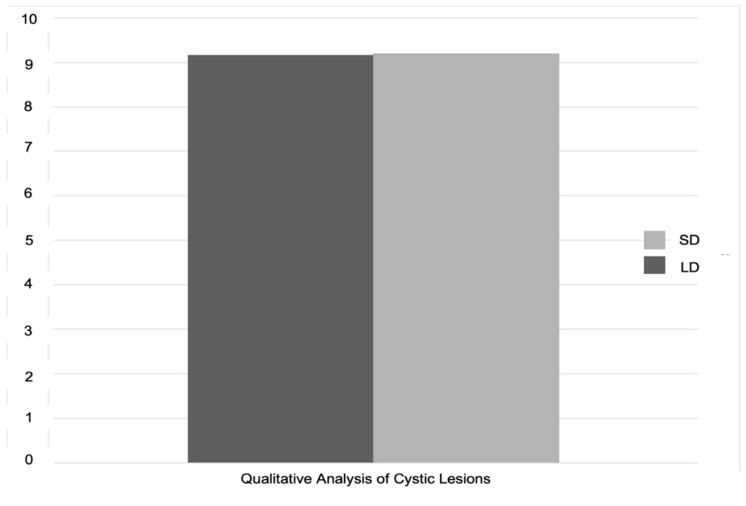
The qualitative analysis of the visibility of cystic lesions according to the dose mode using a scale from 1 (very low) to 10 (very high). Regarding the visibility of cystic lesions, an average value of 9.16 in low dose protocol and 9.19 in standard-dose protocol was registered.

Detection of cystic lesions was successful in 78.8% of cases with the low-dose protocol and in 81.6% with the standard protocol (Figure 2). These percentages are descriptive values obtained from 480 observations resulting from the analyses of all 12 readers. Regarding the visibility of cystic lesions, an average value of 9.16 in low dose protocol and 9.19 in standard-dose protocol was registered (Figure 3).

The model-estimated probability for correct detection (78.8% vs. 81.6% for low-dose and standard-dose) derived from the logistic regression also indicated that lesion detectability did not differ significantly between the two protocols (OR = 0.83, SE = 0.14, *p* = 0.25).

**Figure 4:**
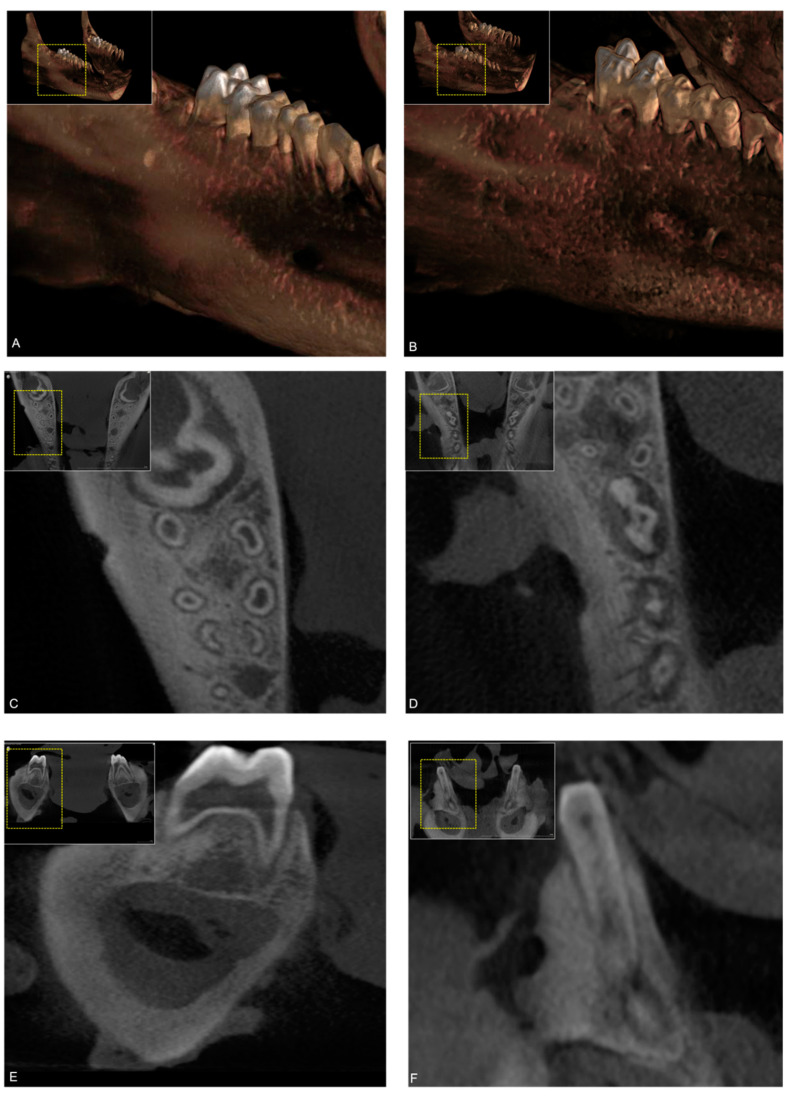
Screenshots of the cystic lesions visualized using the standard-dose (upper row) (image reconstruction (**A**), axial (**C**), and coronal (**E**)) and low-dose (lower row) (image reconstruction (**B**), axial (**D**), and coronal (**F**)) cone-beam computed tomography (CBCT) imaging protocols.

Only minor, non-relevant differences in detectability and visibility were thus observed between low dose and standard-dose imaging protocols (Figure 4).

**Figure 5:**
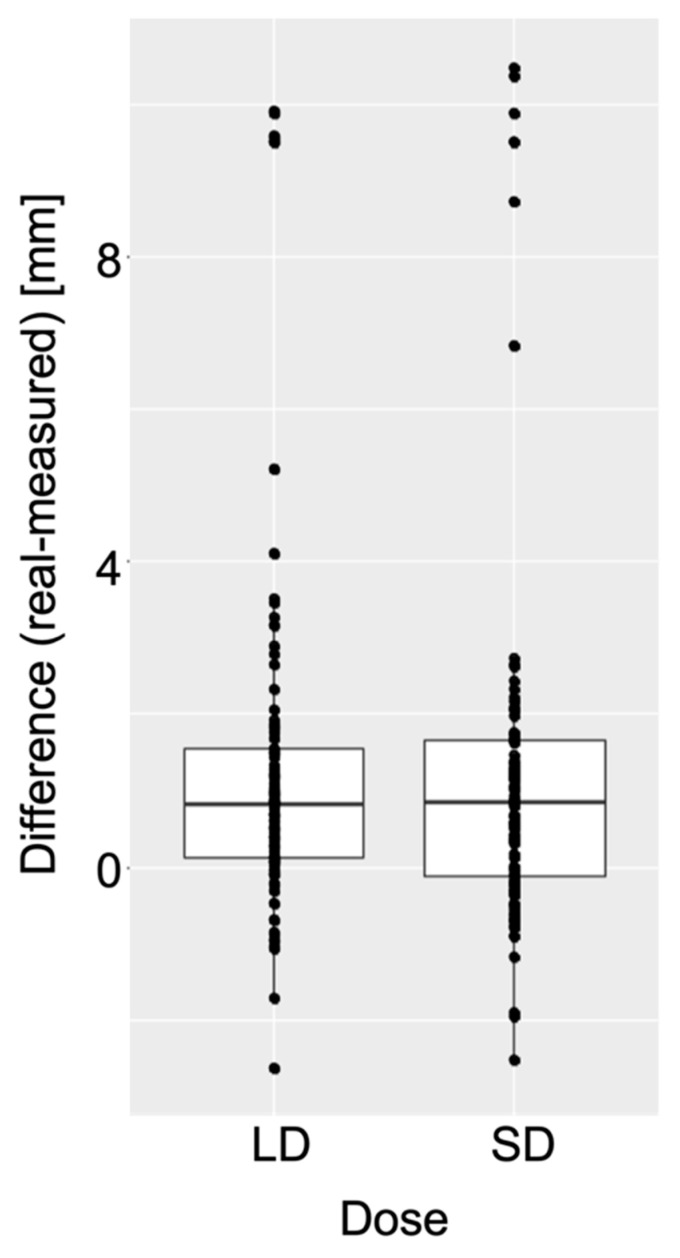
A Wilcoxon signed-rank test with continuity correction was performed based on a significance level of 5% to investigate whether the imaging protocols showed significant differences in their assessment with regard to lesion size. The difference between the measured and actual size of the lesion at its greatest extent in both imaging protocols is visualized. Both imaging protocols were found to perform very similarly with no statistically significant differences (*p* = 0.46) and with an apparent underestimation of the actual distance in both the low-dose and standard protocols.

For all cystic lesions detected by the evaluators in both imaging protocols, the maximum extent of the lesion was measured. In this context, the two imaging protocols were found to perform very similarly, with a general tendency to underestimate the actual distance in both the low-dose and standard protocols by about 1 mm. The Wilcoxon signed-rank test estimated the discrepancy in this difference between the two imaging protocols to be correspondingly small (≤0.3 mm) and showed no statistical significance (*p* = 0.46) (Figure 5).

## 4. Discussion

Considering the revolution in dental imaging over the past few decades and the concomitant increased use of X-ray based three-dimensional CBCT scans in dental surgery and subspecialties, the radiation exposure to the patient should be kept to a minimum without compromising diagnostic accuracy and patient outcomes. In accordance with the ALADA principle, low-dose CBCT protocols have recently been increasingly implemented in clinical routine to improve today’s therapeutic concept of multidisciplinary coordinated, individualized, minimally invasive dental surgery. Since diagnostic reliability compared to standard CBCT protocols has not been fully established, it is important to examine the indications and limitations of each CBCT imaging protocol. These recommendations for the most appropriate application can be implemented into preoperative planning [27]. Therefore, this study aimed to assess the diagnostic reliability of mandibular cystic lesions in vivo using pig mandibles, comparing a pre-set, manufacturer-specific, low-dose mode with a standard-dose CBCT imaging protocol.

Results from a total of 480 cystic lesions examined showed only minor, nonsignificant differences in detectability (78.8% vs. 81.6%) and visibility (9.16 vs. 9.19) between low-dose and standard-dose imaging protocols, with neither imaging protocol differing significantly in lesion size assessment and apparently both generally underestimating actual lesion size. Although attention was paid to accurate preparation of simulated cystic lesions and the elimination of any confounding factors, the overall detectability was around 80%. Compared with previous reports, these in vivo results confirm the detection rate of osseous lesions in porcine mandibles by CBCT reported by Hedesiu et al. and Döbelin et al. of approximately 67.5% to 74.5% and 71%, respectively [30,37].

Although pig models are popular and suitable animal models in orofacial research due to scientific, economic, and ethical reasons and have a high anatomical similarity to the human oral and maxillofacial system, their use is associated with some disadvantages and might explain the lower lesion detectability rate [31,38]. In previous reports, the lower detection rate in ex vivo experiments compared to human studies was explained by the anatomy of the porcine jaw, as it has more anatomical bone holes, more precisely lacunae in the trabecular bone, and variable apical anatomy [37]. In other reports, the preparation methods for the simulated osseous lesions and the macroscopic anatomical differences between porcine and human jaws were mentioned as other possible causes [30].

Given the versatility and increasing demand for CBCT imaging in dentistry, the evidence for indication-specific and patient-specific imaging [39], and the need for radiation dose optimization [40], the primary objective of the current study was to identify potential discrepancies in detection rates between low-dose and standard-dose protocols. The results of this study confirmed the findings of previous studies that both protocols did not show substantial significant differences in the detection of osseous lesions, such as periapical lesion, extended periodontal gap, buccal layer recession, and sequestrum/fracture [30]. Both imaging protocols have observed the underestimation of actual cystic lesion size, whereby recent reports indicate that automated volume determination of osseous lesions is faster but not superior to the manual acquisition, supporting this study’s evaluation method [41]. Nevertheless, accurate volumetric assessment of the mandibular lesions is crucial, as the size of the cyst and its proximity to vulnerable structures, such as the inferior alveolar nerve or adjacent teeth, are essential in choosing the most suitable surgical treatment option for the patient [42,43].

Continuous modernization and optimization of treatment options for jaw cysts remain a challenge in dentoalveolar surgery, with the main surgical procedures used to treat extensive cysts being cystotomy, cystectomy, and two-stage surgery. Indications for marsupialization (cystostomy) are significant loss of lower bone wall, advanced age of the patient, or the presence of severe concomitant diseases; indications for complete enucleation (cystectomy) are small cysts in the area of one to two healthy teeth, extensive cysts if teeth are missing in their area, or sufficient osseous wall thickness [44,45]. Because cystic lesions are often discovered as asymptomatic incidental findings on panoramic radiographs, three-dimensional imaging techniques remain of elementary importance to provide multiplanar cross-sectional and three-dimensional reconstructions for further diagnosis and preoperative treatment planning [46,47].

In the low-dose protocol used in our experimental setup, halving the radiation time (LD = 2.2 s; SD = 4.4 s) and the use of a larger copper diaphragm (LD = 1 mm copper diaphragm; SD = 0.3 mm copper diaphragm) resulted in an effective radiation dose reduction of 85% (LD = 20 mSv; SD = 145 mSv) [32]. Thus, three-dimensional information, particularly needed for repeated radiographic evaluation of cysts with recurrence tendency, can be obtained with radiation exposure comparable to that of conventional imaging techniques such as panoramic radiography [48,49]. Considering that radiation-induced cancer risk is cumulative throughout life and the increasing use of CBCT in the radiation-sensitive population of young adolescents, this is another step toward minimizing or, in the further future, even eliminating radiation without substantial loss of diagnostic accuracy [23,25]. As mentioned, low-dose imaging protocols can be achieved through a variety of modifications, such as reducing tube current (mA), resolution, scan time, use of partial rotations, and number of projections [50]. Thereby, the FOV can also be a key factor, as it can contribute significantly to effective radiation dose reduction. The choice of a small FOV (4 × 4 cm) compared to the largest FOV (17 × 12 cm) is associated with a dose reduction of approximately 80% [51]. As an extended approach to radiation dose optimization, adjusting the tube voltage (kV) and determining the minimum acceptable tube current (mA) can reduce the dose used in standard protocols by 40% [8]. These statements are supported by the results obtained within this study, as the applied low-dose protocol allowed no relevant impairment in the detectability of cystic lesions.

There are several limitations in this study. Firstly, there is a limitation due to use of porcine mandibles. Thus, it is challenging to make clinically relevant conclusions. Therefore, further human studies are needed to confirm the trends obtained. Second, the assessment method may have influenced the results, as a learning effect could have occurred, leading to improved performance in image analyses performed later. However, it has been shown that after brief training, results are obtained that show a tendency toward reliability independent of the reader’s experience. Nevertheless, further studies are required to assess accurate intra- and inter-reader reliability. Thirdly, the experience of the investigators might have had an influence. Nevertheless, no apparent differences were observed between the readers. This fact could allow the introduction of this imaging modality into daily clinical routine for general dentists who are less experienced in radiographic assessment of CBCT imaging.

From a clinical perspective, low-dose protocols for CBCT imaging are a promising imaging modality in various dental disciplines such as pediatric dentistry, orthodontics, endodontics, and oral and maxillofacial surgery. Since there are no guidelines for using low-dose protocols and due to the implementation variability regarding scanners and scanning parameters, further studies should be conducted with well-designed protocols to better understand indications and limitations. Given the heterogeneity of device-specific protocols, research is needed to evaluate reliability with other devices and further develop standardized protocols that can be applied independently of manufacturer-specific settings. Nevertheless, PAN and single-tooth radiography are and will remain an essential part of primary diagnosis. Alternative imaging modalities associated with less or no radiation dose, such as ultrasound or magnetic resonance imaging, should also be considered when appropriate [15]. In summary, however, further improvements and standardization of low-dose CBCT protocols are needed in terms of consistency and discriminability, taking into account relevant scientific, economic, and ethical factors that can be translated into personalized therapy.

## 5. Conclusions

The use of low-dose CBCT protocols provided a confidential and reproducible radiographic assessment of cystic lesions, considering detectability rate, visibility, and size assessment, without any relevant differences compared to standard CBCT protocols. After further validation of these data in human studies, the targeted use of low-dose CBCT protocols could be introduced into future routine clinical practice as a primary diagnostic tool and, more importantly, as an imaging modality for postoperative follow-up, enabling postoperative care with an improved benefit-risk ratio.

## Figures and Tables

**Table 1:** This table shows the settings of the low-dose and standard-dose cone-beam computed tomography (CBCT) protocols of the Orthopos SL (Dentsply Sirona, York, PA, USA) dental X-ray imaging unit.

Mode	FOV (cm)	kV/mA	Radiation Time (s)	Voxel Size (µm)	Effective Dose (µSv)
LD	11 × 10	85/13	2.2	160	20
SD	11 × 10	85/13	4.4	160	145

## Data Availability

The data presented in this study are available on request from the corresponding author. The data are not publicly available.
